# Transport and instream removal of the *Cry1Ab* protein from genetically engineered maize is mediated by biofilms in experimental streams

**DOI:** 10.1371/journal.pone.0216481

**Published:** 2019-05-16

**Authors:** Arial J. Shogren, Jennifer L. Tank, Emma J. Rosi, Martha M. Dee, Shannon L. Speir, Diogo Bolster, Scott P. Egan

**Affiliations:** 1 University of Notre Dame, Department of Biological Sciences, Environmental Change Initiative, Notre Dame, Indiana, United States of America; 2 Cary Institute of Ecosystem Studies, Millbrook, NY, United States of America; 3 University of Notre Dame, Department of Civil and Environmental Engineering and Earth Sciences, Notre Dame, Indiana, United States of America; 4 Rice University, Department of BioSciences, George R. Brown Hall, Houston TX, United States of America; University of Nantes, FRANCE

## Abstract

The majority of maize planted in the US is genetically-engineered to express insecticidal properties, including *Cry1Ab* protein, which is designed to resist the European maize borer (*Ostrinia nubilalis*). After crop harvest, these proteins can be leached into adjacent streams from crop detritus left on fields. The environmental fate of *Cry1Ab* proteins in aquatic habitats is not well known. From June-November, we performed monthly short-term additions of leached *Cry1Ab* into four experimental streams with varying benthic substrate to estimate *Cry1Ab* transport and removal. At the start of the experiments, when rocks were bare, we found no evidence of *Cry1Ab* removal from the water column, but uptake steadily increased as biofilm colonized the stream substrate. Overall, *Cry1Ab* uptake was strongly predicted by measures of biofilm accumulation, including algal chlorophyll *a* and percent cover of filamentous algae. Average *Cry1Ab* uptake velocity (v_f_ = 0.059 ± 0.009 mm s^-1^) was comparable to previously reported uptake of labile dissolved organic carbon (DOC; mean v_f_ = 0.04 ± 0.008 mm s^-1^). Although *Cry1Ab* has been shown to rapidly degrade in stream water, benthic biofilms may decrease the distance proteins are transported in lotic systems. These results emphasize that once the *Cry1Ab* protein is leached, subsequent detection and transport through agricultural waterways is dependent on the structure and biology of receiving stream ecosystems.

## Introduction

Up to 80% of all maize planted throughout North America is now genetically engineered (GE) to express one or more insecticidal proteins to combat targeted agricultural pests (NASS 2016). For example, one of the most common varieties of GE maize is designed to resist crop damage by expressing *Cry* defensive proteins. While these GE derived proteins include a variety of *Cry* proteins, here we focus on a specific variant derived from a bacterium (*Bacillus thuringiensis*), hereafter referred to as Bt maize, which is used to combat damage caused by the European maize borer (*Ostrinia nubilalis*). Like many GE-derived defensive proteins, the *Cry1Ab* protein is expressed throughout the entirety of the Bt maize plant tissue, including leaves, roots, stalks, and pollen [1], and the effects of direct exposure to *Cry1Ab*-containing maize detritus on non-target terrestrial organisms have been well documented [2,3]. Several studies have also reported potential deleterious effects of Bt maize on non-target aquatic invertebrates [3,4], and Bt maize inputs of *Cry1Ab*, or other defensive GE-proteins, may be an additional stressor on aquatic ecosystem structure and function in already degraded agricultural streams [4]. The widespread planting of Bt maize across the agricultural Midwest has raised questions regarding the fate and potential ecological effects of GE-proteins contained in crop residues entering the environment, particularly for adjacent waterways [5].

There are several critical input pathways for Bt maize detritus to enter adjacent waterways, and of particular concern is the continuous deposition of Bt maize detritus from crop detritus after harvest via no-till agricultural practices and their subsequent delivery to adjacent surface waters via leach and wind erosion [3–8]. Inputs of Bt maize detritus in agricultural streams can result in elevated organic matter standing stocks, with previous studies recording up large standing stocks of *Cry1Ab*-containing maize material per m^2^ of streambed [4]. Once Bt maize detritus enters a stream, the material can be retained, decomposed, or consumed *in situ*, or the detritus can be distributed throughout stream networks, far from the source of entry, especially during high flows [5]. In addition to the direct inputs of particulate Bt maize detritus from fields into adjacent waterways, the Cry proteins also leach quickly from detritus, either into soils, or into waterways directly via stream submersion of detritus [9,10]. A series of laboratory experiments demonstrated that when Bt maize material is submerged, >90% of the initial *Cry1Ab* protein leached after only 24 hrs, and a mere 1% of the protein remained in source detritus (i.e., maize leaves) after 70 d [6]. Transport of leached proteins from Bt maize detritus via overland flow [11], subsurface tile drainage [10], or via groundwater [12] can result in sustained inputs of *Cry1Ab* into adjacent ditches and may contribute to the ubiquitous detection of *Cry1Ab* protein in water samples collected from agricultural streams across space and time (average *Cry1Ab* concentration = 32 ng L^−1^; [5]).

The mechanisms controlling *Cry1Ab* detection and transport through watersheds are not well known, which underscores the importance of identifying the ultimate fate of leached proteins and isolating the conditions that mediate their transport or removal throughout stream networks. In this study, we used a combination of field experiments and modeling scenarios to examine the influence of stream bottom (i.e., benthic) substrate and associated biofilms on the removal of leached *Cry1Ab* from stream water. To simulate conditions typifying a biofilm colonization sequence, we performed monthly short-term additions of leached *Cry1Ab* from June-October in low-gradient open-canopy experimental streams, and finally ending in November with a simulated disturbance event resulting in biofilm scouring. Our experiments represented conditions typical of Midwestern agricultural streams, from the crop *growing season* through *post-harvest* with increasing biofilm colonization under high light and warm temperatures, and then ending with a major *disturbance* (e.g., storm event in November) that reduced instream biology. Given our highly controlled field setting, we had a unique opportunity to isolate the influence of biofilm colonization and substrate on the instream removal of leached *Cry1Ab*. Additionally, while disturbance can often confound results in field studies [13], our experimental streams allowed us to examine uptake dynamics under controlled flow conditions, and also following an experimental disturbance. Our goal was to use simple empirical and modeling tools to assess the effects of substrate type, biofilm colonization, and disturbance on stream processing of a GE-derived Cry-protein, to improve our understanding of controls on it fate in agricultural streams.

We used four unique, controlled streams at the Notre Dame Linked Experimental Ecosystem Facility (ND-LEEF) as a platform to explore the potential drivers of *Cry1Ab* removal from flowing waters. The streams at ND-LEEF differed only in the substrate size/orientation lining the stream bottom. Based on previous experiments performed at ND-LEEF, substrate size influenced the typology and trajectory of biofilm colonization, with larger substrates (e.g., cobble) promoting increased biofilm accumulation. In turn, biofilm colonization mediated the transport of conservative tracers [14]. We hypothesized that leached *Cry1Ab* proteins would act similar to the transport of labile dissolved organic carbon (DOC), and thus we expected that transport would vary over the growing season, with biofilm accumulation resulting in increased removal of *Cry1Ab* from the water column. Conversely, we predicted that biofilm scouring during a disturbance event (e.g., storm) would decrease *Cry1Ab* removal and promote downstream transport given reduced biological complexity.

## Methods

### Site description

**Fig 1:**
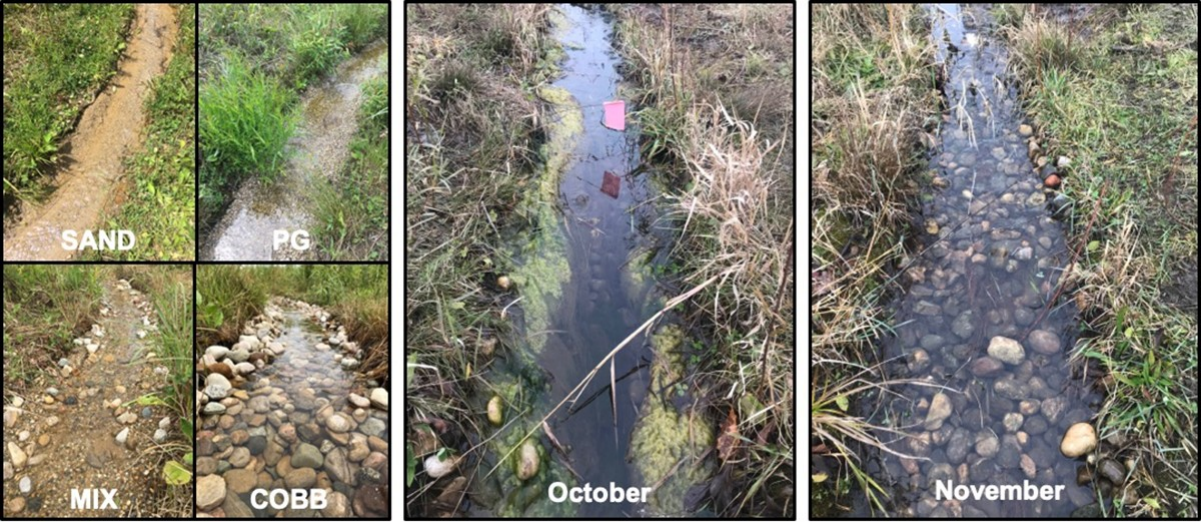
ND-LEEF streams before, during, and after colonization. Picture panel of streams at ND-LEEF for A) on Day 0, B) during peak biofilm colonization in COBB showing the dominant cover of filamentous green algae, and C) post-disturbance in COBB. Key: PG = pea gravel, COBB = cobble. Pictures were taken by AJS, SLS, and MMD.

We conducted this study in four experimental streams at the ND-LEEF field site located in South Bend, Indiana (41.67° N, 86.25° W) in partnership with St. Patrick’s County Park; each stream is 50 m long, 0.4 m wide, and concrete-lined to isolate systems from groundwater exchange. For our experiments, stream discharge was held constant at ~0.9 L s^-1^ throughout the growing season to simulate baseflow conditions during a typical summer growing season. To examine the effects of substrate complexity on *Cry1Ab* transport and removal, we lined each stream with a unique configuration of benthic substrate (Fig 1): sand (SAND, D_50_ = 2 mm), pea gravel (PG, D_50_ = 0.5 cm), cobble (COBB, D_50_ = 5 cm), and an equal mix of all substrate sizes (MIX). All substrate was bare at the start of the experiment (Day 0 in June), and the streams colonized by biofilm without disruption from June through October, during which time we conducted monthly experimental *Cry1Ab* releases to capture a typical biofilm colonization sequence. At the end of the experiment, after 159 days of undisturbed biofilm growth, we performed a manual sloughing event to remove all biofilm from the benthic substrate in each stream, then repeating the experimental *Cry1Ab* release for a “post-disturbance” measurement. We recognize that stream substrate treatments were not replicated in this study. Our field site has only four stream reaches, limiting the strength of our statistical findings.

### Physical characteristics

We measured a variety of physiochemical variables to assess the relationship between biotic and abiotic stream characteristics and subsequent *Cry1Ab* removal. On each sampling date, we measured wetted stream width (*w*, m) and depth (*d*, m) as the average of 10 measurements taken along each stream reach. Additionally, on every date we estimated water velocity (*v*, m s^-1^) and discharge (*Q*, L s^-1^) using conservative tracer additions of sodium chloride (NaCl; [15,16]). Before each experimental *Cry1Ab* release, we collected benthic samples for estimation of 1) algal biomass using chlorophyll *a* (chl a) and 2) organic matter (OM) as ash-free dry mass (AFDM) every 10 m along each 50 m reach for each stream (n = 4). For chlorophyll *a*, we collected ~100 mL of streambed substrate in clean 160 mL specimen cups, carefully draining any stream water from the sample (n = 5 per stream per sampling date). We immediately froze each sample until pigment analysis. After briefly thawing each sample, we extracted chlorophyll *a* from each sample using the cold-methanol fluorometric method with a ~24 hour extraction time, with samples extracting overnight in a fridge [17]. We measured the extract on a fluorometer [18], and compared the sample absorbance with a 6-point standard curve dilution (from 0 to 500 mg chlorophyll a L^-1^). We expressed chlorophyll as mass per volume (mg chlorophyll a L^-1^). We then scaled each chl a measurement for surface area and expressed replicates in mg chl a cm^-2^ of streambed. We also estimated benthic OM by placing subsamples of each substrate type (n = 5 per stream per day) in ashing tins, dried for 48 hours at 60°C, and weighed after drying to obtain dry mass. We then ashed the samples at 550°C for 1 hr and reweighed the samples. We calculated AFDM as the difference between the dry weight and ashed weight of each subsample, and divided this value by the subsample surface area (38.4 cm^2^) to get OM expressed as mg AFDM cm^-2^ of streambed surface [19].

To capture the spatial heterogeneity of algal and biological colonization, on each sampling date we estimated the percent cover of filamentous green algae, terrestrially-derived organic matter, and benthic algal biofilm along 10 lateral transects in each stream. In our visual survey of stream biology, “filamentous green algae” were categorized as attached periphyton strands that extended >1 cm into the water column and could include periphyton biomass that accumulated at the water surface [20]. Benthic biofilms, in contrast, were classified as attached biofilms that lined the substrate surface but did not protrude into the water column. While we did not separate these algae taxonomically, these categories were meant to describe the majority of biofilm and algae occupying the stream channel. Both the cover of biofilm mats [21] and periphyton structure [20] have been shown to influence particle deposition rates. Finally, we estimated the total OM standing stock of encompassed by biofilm, benthic OM, and filamentous green algae at the termination of the experiment, measured as the total mass of material, removed from each stream after the final disturbance event, using manual collection as well as nets placed at the bottom of each 50 m reach.

To quantify changes in stream metabolism over the biofilm colonization sequence, we deployed miniDOT loggers (PME, Inc., CA, USA) at the top and bottom of each stream reach for the entire field season. Each miniDOT logger collected dissolved oxygen (O_2_) and water temperature, recorded every 10 min. For each sampling day, we estimated reach-scale gross primary productivity (GPP), ecosystem respiration (ER) and gas exchange rate (K) for the 3-day period encapsulating each sampling date using the two-station, open-channel exchange method [22]:

$${oxy}_{down(t)}=\frac{{oxy}_{up(t)-lag}+[\frac{GPP}{z}*\frac{\sum_{t+lag}^{t}PAR}{{PAR}_{total}}]+\frac{ER*tt}{z}+K*tt*\frac{\left({oxy}_{up(t),sat}-{oxy}_{up(t)}+{oxy}_{down(t),sat}\right)}{2}}{1+\frac{K*tt}{2}}$$

In this model, *oxy*_*down(t)*_ is the downstream O_2_ concentration at time t (g O_2_ m^-3^); oxy_up(t)—lag_ is upstream O_2_ concentration of the same parcel of water after a lag period corresponding to travel time, tt (d^-1^); z is mean stream depth (m); *ΣPAR* is the sum of photosynthetically active radiation (PAR) at time t through the lag period (μmol m^-2^ s^-1^); *PAR*_*total*_ is the cumulative PAR for the entire period; K is the air-water exchange rate for oxygen at time t, and oxy_up,down(t),sat_ is the saturated concentration of oxygen (g O_2_ m^-3^) at time t based on temperature and barometric pressure. For each model, we used K_600_, which is a metric comparable across the range of temperatures, by normalizing K based on a Schmidt number scaling [22]. As a quality control check, we compared model estimated K values on each date to empirically measured gas reaeration coefficients conducted at ND-LEEF at similar times (Dee, *unpublished data*). We expressed both GPP and ER in g O_2_ m^-2^ d^-1^, and denoting GPP as positive (i.e., production) and ER as negative rates (i.e., consumption) of O_2_ change. The model assumes that GPP is a linear function based on light availability, thus GPP is driven by the amount of PAR attenuated by the water in each time interval (t to t+lag). We obtained all PAR and barometric pressure data for each sampling date from a weather station deployed at the ND-LEEF field site (EmNet, LLC, South Bend, IN).

### Confirming Cry1Ab presence before leaching

We collected Bt maize leaves and stalks from the upland slopes of streams and ditches in northwest Indiana in October 2016, when maize material typically enters systems from surrounding agricultural fields, which is typical for this Midwestern region. We dried maize material at room temperature and stored material in plastic bins prior to experimental leaching. We tested subsamples of dried maize leaves from each bin to confirm the presence of the *Cry1Ab* protein using commercially available Agdia ImmunoStrip detection strips for Bt-*Cry1Ab/1Ac* (Catalog No: STX 06200). When positive for *Cry1Ab/1Ac*, a test line appears on the strip, confirming expression of the protein. All maize material used to generate the release solution for the experimental additions tested positive for the Bt-*Cry1Ab/1Ac* protein. We did not directly measure protein degradation directly during storage, and cannot rule out the possibility that concentrations may have declined between detritus collection in the field and processing on ELISA. However, we used published storage methods for *Cry1Ab* in both dried maize material and in the collected stream water samples [4,5,10,23], and processed the samples on ELISA within 3 weeks of each experiment. Therefore, any issues from storage should have been minimized, and comparable to any other studies measuring *Cry1Ab* in stream water.

### Short-term Cry1Ab additions

To conduct short-term *Cry1Ab* releases, we modified standard methods of short-term nutrient additions commonly used to quantify uptake [16]. For each stream, we used 5 longitudinal sampling locations for the entire 50 m reach, thus sampling every 10m from the release site. Before the start of each experiment, we collected ~40 mL “background” water samples to determine any ambient *Cry1Ab* in stream water, and measured background conductivity using a Hydrolab Minisonde (Hach, Loveland, CO). Immediately prior to each release, we added 200 g NaCl to the release solution as a conservative tracer. We used the conservative tracer both to determine when the streams were at a well-mixed “plateau”, and to calculate stream discharge via dilution gauging [15]. First, we measured stream conductivity before and during each experimental release using a calibrated Hydrolab Minisonde (Hach, Loveland, CO). We used the conductivity before (i.e., “background”) and during the well-mixed “plateau” to determine when sampling was appropriate [15,16]. We dripped the release solution into each of the four streams at a constant rate of 100 mL min^-1^ using a peristaltic pump (Fluid Metering Incorporated) until each stream reached “plateau” condition, as reflected by steady but elevated specific conductance at all stations within each reach. After the streams had reached the plateau stage, at each downstream station we took three replicate water samples to estimate *Cry1Ab* concentration (n = 15 samples per stream). To quantify the mass of *Cry1Ab* added to the streams during each experiment, we took an additional sample from the release solution bucket during each release experiment. We immediately syringe-filtered each sample (40 mL) using 25 mm 0.7 um GF/F filters (Whatman), then stored samples on ice until returning to the lab, where samples were frozen at -4°C until processing and quantification.

Lastly, we estimated water velocity (*v*, m s^-1^) and discharge (*Q*, L s^-1^) using a standard dilution gauging technique, where discharge is estimated by the dilution of the tracer at the downstream sampling point [24]. In this method, we assume that there is little-to-no loss of NaCl from sorption and that the solution is not affecting ecological function. We designed our experiment to only increase conductivity from background by ~5% (~20–50 μS cm^-1^). Given the high background conductivity in our streams (>500 μS cm^-1^), our additions should not have impacted stream function.

### Simulating a stream disturbance event

From June-October, biofilm was allowed to colonize each stream naturally, and because flow maintained throughout the period, biofilm was not disturbed or sloughed from the benthic substrate, resulting in significant accumulation of substrate biofilm as well as OM in substrate interstices. To parse out the role of organic matter and biofilms on *Cry1Ab* removal, we performed a thorough sloughing event in November by manually removing all biofilm and disturbing accumulated streambed OM. Our manual sloughing event involved a team of 4 people, moving in a line from upstream to downstream removing mats of filamentous green algae, terrestrial organic matter and roots, and overturning the substrate by hand, using rakes, and mobilizing the substrate with boots. To maximize biofilm and organic matter removal, we performed at least three slough passes in each stream. Additionally, after each manual slough, we increased the stream flow rate (~4–5 L s^-1^, 5x baseflow) for ~1hr to flush remaining finer materials from the stream. After the slough, we returned the streams to baseflow and allowed the streams to settle for ~24 hrs until our experimental releases. We then repeated the experimental *Cry1Ab* addition for a “post-disturbance” measurement in each stream. To quantify total OM in each stream, we collected all filamentous algae and larger OM by hand (pooling in 10 m increments) placing in bags for later estimation of total biomass for each stream reach. Later, in the laboratory, we dried the OM in subsamples and estimated standing stocks per stream (g dry mass). We repeated sampling for chl a, benthic OM, and substrate transects immediately after the disturbance event to confirm sloughing.

### Analysis of Cry1Ab protein in stream water samples

In order to measure low concentrations of *Cry1Ab*, we concentrated each sample using Amicon Ultra-15 mL centrifugal filter units (30K Nominal Molecular Weight Limit, Millipore). We followed the centrifugal extraction method and subsequent Enzyme-Linked ImmunoSorbent Assay (ELISA) assay to measure *Cry1Ab* in stream water using field-validated techniques that have shown high recovery of Cry protein using phosphate buffered saline with tween buffer (PBST; [10,11]). Briefly, to extract the protein from each water sample, we pipetted 14.5 mL of stream sample into the Amicon filter units and added 0.5 mL 1X PBST buffer. We separated the retentate from supernatant using a centrifuge with 60 mL centrifuge tube adapters at 2500 RPM for 30 minutes. The remaining concentrated *Cry1Ab* retentate was immediately pipetted into a sterile microcentrifuge tube.

After extraction, we quantified *Cry1Ab* protein in water samples using a commercial double-antibody sandwich ELISA (Agdia, Elkhart, IN, Part No: 06200/0096) as described by Strain and others [11]. For each sample, we pipetted 100 μL of the sample, standard, and blank (3 DI water, 3 PBST) retenate in triplicate onto 96-well ELISA plates. After following the manufacturer protocol, we read the plate absorbance at both 450 nm and 650 nm on a SpectraMax M5 microplate reader (Spectra Max M5, Molecular Devices, CA, USA). To correct for any turbidity in our samples, we then subtracted the resulting absorbance at 450 nm from the absorbance read at 650 nm. To account for any matrix effects of the PBST, we subtracted the mean PBST blank absorbance from the (650-450nm) estimation. Because we wanted to determine relative concentration of the protein in our sample, expressed in ng *Cry1Ab* L^-1^ stream water, each plate included a five-point calibration curve created from a serial dilution of purified *Cry1Ab* (MyBioSource, Part No: MBS537737) dissolved in DI water [11]. The calibration curve ranged from 2 ng L^-1^ to 400 ng L^-1^ *Cry1Ab*, and the curve was run in duplicate on each ELISA plate.

### Calculating Cry1Ab uptake metrics

On each sampling date, in each of the four streams, we used the decline in *Cry1Ab* concentrations to estimate a Cry protein uptake length (*S*_*w*_, m), which is a quantitative metric representing the average distance a *Cry1Ab* protein travels in the water column before being physically or biologically retained [16,25]. When mass is lost into the stream, the steady input of Cry1Ab into each stream results in an exponential decrease in concentration with downstream distance relative to the site of addition. Using dilution-corrected concentration data for *Cry1Ab*, we fit the longitudinal mass lost from each experimental release to the first-order equation: ln *N*_*x*_ = ln *N*_0_ –*kx*. The terms *N*_0_ is the initial concentration of Cry1Ab that is released, and the *N*_*x*_ term is the *Cry1Ab* subsequently passing over each station (n = 5) *x* m downstream after the stream is well-mixed. In this equation, *k* represents the per m removal rate over reach distance. Thus, the inverse of this term (*k*^-1^) represents the average distance that a protein will travel before being deposited onto the streambed and removed from transport, which is also denoted as uptake length *S*_*w*_ (in m; Stream Solute Workshop, 1990). However, the *S*_*w*_ metric is strongly influenced by flow, which may vary among streams [26,27]. Therefore, we converted the *S*_*w*_ metric to an uptake velocity (*v*_*f*_), that represents relative demand, and this metric enables comparison among streams and with previously published values. We calculated *Cry1Ab v*_f_ (mm s^-1^) = (*Q*/*w)/S*_*w*_, where *Q* is discharge (L s^-1^), *w* is wetted channel width (m). The metric *v*_f_ represents the velocity at which *Cry1Ab* moves from the water column to the benthos [16]. Importantly, both uptake length (S_w_) and uptake velocity (v_f_) represent reach-scale processes, which can include a combination of biological and physical processes that temporarily, or permanently, retains or removes *Cry1Ab* protein from the flowing stream water.

### Statistical analysis

First, we assessed the trajectory of biofilm metrics in each stream over time. We fit Linear Models (LM) to compare biofilm accumulation over time for chlorophyll a and organic matter. We compared streambed biofilm metrics on each sampling date using one-way analyses of variance (ANOVAs) and Tukey’s HSD post-hoc test to evaluate significant differences among streams. We tested data for assumptions of Normality (Shapiro-Wilks test) and constant variance (Bartlett test), and log-transformed the data when necessary (summary statistics in S1 Table).

Then, we estimated *Cry1Ab* removal rates in each stream on each sampling date and over time, and investigated potential drivers of measured rates. We calculated *Cry1Ab* uptake metrics only from releases with significant uptake regressions (i.e., R^2^ > 0.8, p < 0.05). On each individual sampling date, we tested for substrate-driven differences by comparing *Cry1Ab* uptake metrics across stream substrate types and used the interpretation of an analysis of covariance (ANCOVA) to compare the slopes of the decline in *Cry1Ab* along each stream reach; this allowed us to determine differences in uptake among stream substrate treatments on each sampling date, and the approach has been used for decomposition [28] and nutrient uptake studies [27]. To test whether there was a difference in *Cry1Ab* uptake velocities over time, we used rmANOVA. Then, we examined temporal trends of *Cry1Ab* uptake using linear models of v_f_ over time; for each metric, we determined the best model (linear or quadratic) based on R^2^. Finally, we determined the variables controlling *Cry1Ab* uptake using Pearson’s Correlation and by conducting stepwise multiple linear regression (MLR) analyses. We ran all statistics using R Studio (version 1.1.383).

## Results

### Biofilm characteristics

**Fig 2:**
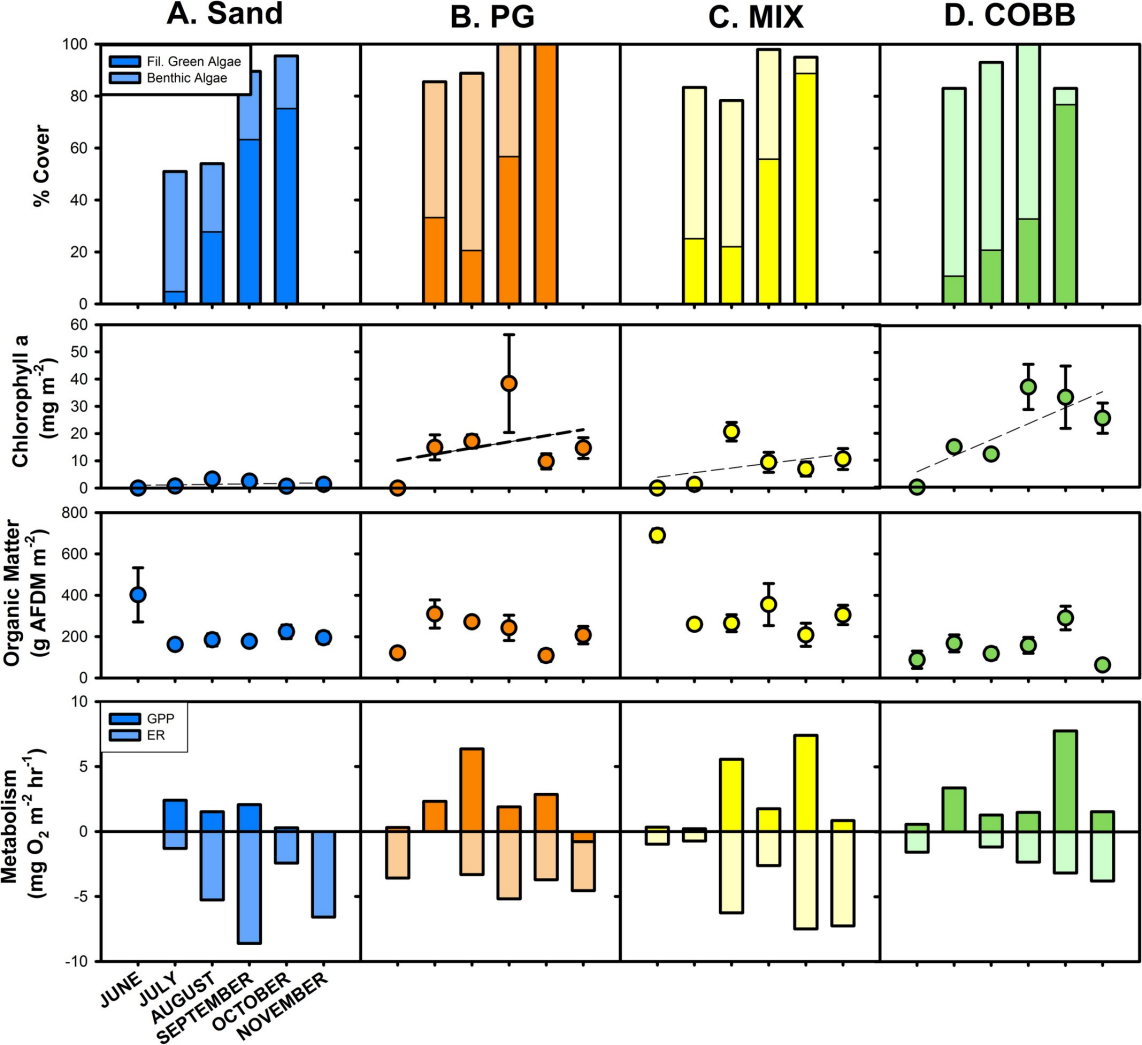
ND LEEF stream biofilm. Biofilm metrics among streams for: percent cover Filamentous Green Algae and Benthic Algae (top row); average (± SE) Chlorophyll a (second row); average (± SE) benthic OM (third row); and modelled metabolism metrics, GPP and ER (bottom row) from ND-LEEF streams across sampling dates. Dashed regression lines indicate a significant linear regression (at p < 0.05).

Each stream revealed a unique biofilm colonization trajectory, using chlorophyll a and organic matter as proxies for algal growth (Fig 2). There was no visible biofilm or measurable chl a present in the streams when flow was turned on at the start of the experiment (June), after which benthic algae rapidly colonized each stream. Biofilm chl a (mg m^-2^) increased from June through September, generally peaked in August for SAND and MIX, and in September for PG and COBB (rm ANOVA, p<0.05) (Fig 2). Across all sampling dates, chl a was consistently higher in PG and COBB than in MIX or SAND (ANOVA followed by Tukey HSD, p < 0.001, Fig 2). Results for organic matter (OM) were highly variable across streams and over time.

In conjunction with patterns of biofilm accumulation indicated by chl a and benthic OM, we also observed changes in algal composition as percent cover. Total algal cover (benthic algae and filamentous green algae) increased from 0% on Day 0 to between 20–80% of the substrate surface after 30 d and covered 100% of the substrate surface when biofilm colonization peaked in August/September (Fig 2). During initial colonization (July-September), benthic algae was generally the dominant cover in each stream, and average % cover across all sampling stations ranged from 40–80% in COBB, 20–60% in MIX, 40–80% in PG, and 20–50% cover in SAND. In these same months, average % cover of filamentous green algae ranged from 8–28% in COBB, 22–58% in MIX, 28–57% in PG, and <10–54% in SAND. However, after the initial colonization period from July through September, filamentous green algae gradually became the dominant cover (Fig 2), peaking in October, at 76% in COBB, 78% in MIX, 90% in PG, and 62% in SAND. An increase in filamentous green algae coincided with a decrease in benthic algal cover, which ranged from 0–30% cover for all streams after September. After the disturbance event, % cover of benthic and filamentous green algae was negligible in all streams.

We measured the total biomass removed from each stream as a result of the disturbance event and documented remaining biomass in each stream after the event. Biomass removed during the disturbance event was dominated by filamentous green algae and the total dry mass (DM) measured was 1.4 kg DM from COBB, 3.93 kg DM from MIX, 7.0 kg DM from PG, and 7.1 kg DM from SAND, with biomass being generally highest in the first 20 m of each stream reach.

### Instream function

As biofilm biomass increased over time, GPP and ER showed distinct temporal trends in each stream over the trajectory of biofilm colonization (Fig 2). Overall, we found no statistical differences in GPP among streams (rmANOVA, p > 0.05), and GPP was generally lowest in all 4 streams in June and after our disturbance in November. However, across sampling dates from June-October, GPP was lowest in PG and SAND, and highest in COBB and MIX (Fig 2). There was an initial increase in PG and MIX such that we measured peak GPP in August in both streams, while GPP peaked in July in SAND and COBB. We observed similar trends for ER, although the range rates within and among streams was larger than for GPP; ER was generally highest in PG and COBB, and lowest in SAND and MIX. Similar to GPP, we found no statistical differences in ER among streams (rmANOVA, p>0.05), although COBB and PG tended to have higher ER than MIX and SAND. We also saw a significant decrease in ER post-disturbance (ANOVA, p < 0.05), confirming that we successfully decreased biofilm biomass and reduced biological function in all streams with the disturbance.

### Does benthic substrate influence Cry1Ab uptake in streams?

**Fig 3:**
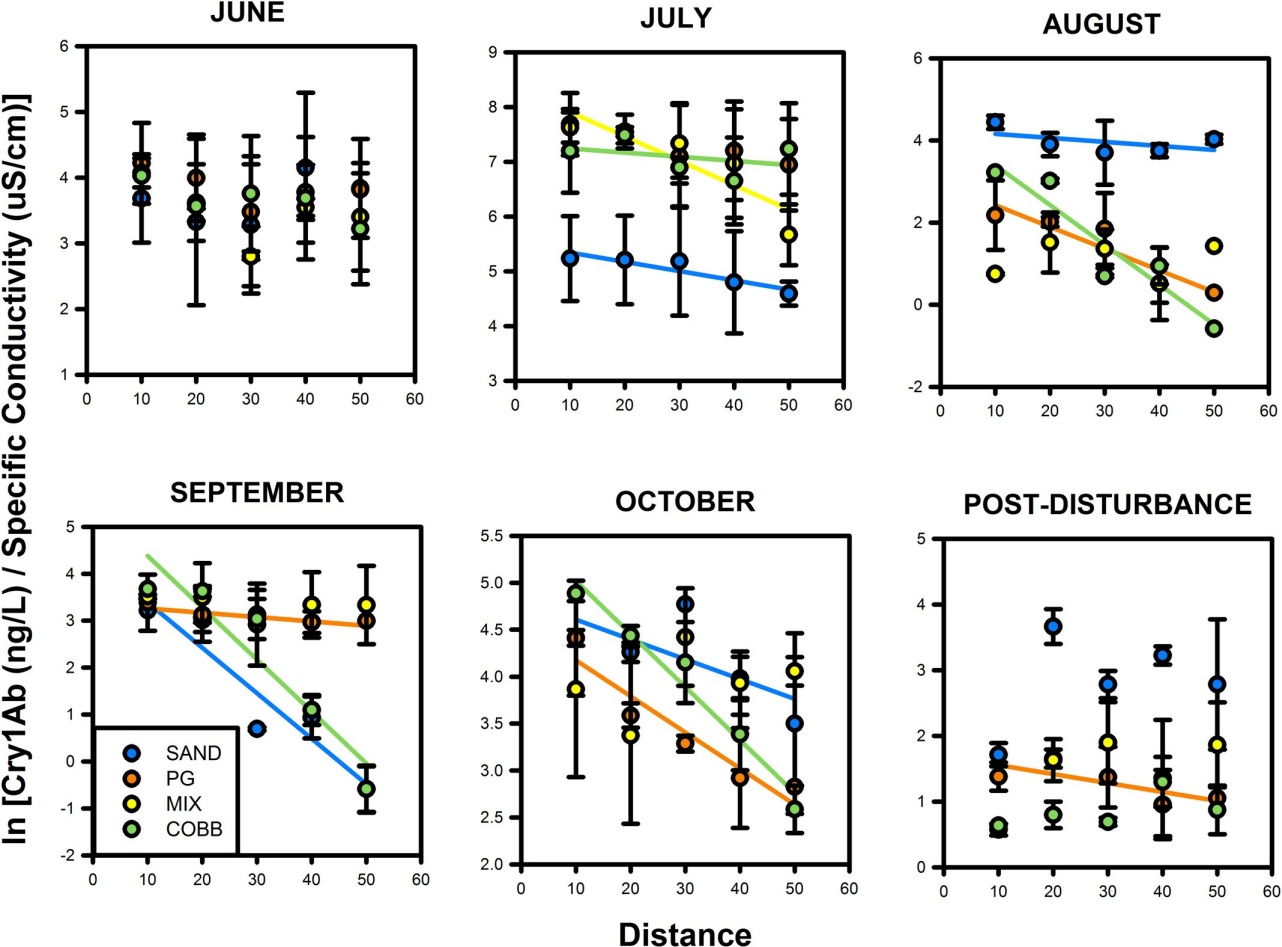
Cry1Ab release regressions. Dilution-corrected declines in *Cry1Ab* concentration over distance in ND-LEEF streams over the biofilm colonization sequence. Regression lines indicate significant relationship (R^2^ > 0.8, p < 0.05.

For each of the 24 releases we conducted on 6 sampling dates, we report *Cry1Ab* uptake (i.e., removal from the water column) as a longitudinal uptake rate (the slope, *k*) for all releases with statistically significant decline in dilution-corrected *Cry1Ab* concentrations over the stream reach (Table 1, Fig 3). On the June sampling dates, there was no significant *Cry1Ab* uptake; however, substrate significantly influenced *k* during July, August, and September samplings (ANCOVA: Stream x Distance, p < 0.05). While each substrate treatment influenced *Cry1Ab k* during these sampling months, patterns were not consistent by substrate, but rather followed that higher instream biofilm resulted in higher *k*. The effect of substrate on *k* was not significant during the sampling dates later in the field season (October; ANCOVA: Stream x Distance, p > 0.05 *for all*), and we only observed significant regressions in one stream (PG) after the disturbance.

**Table 1:** *Cry1Ab* tansport and removal metrics for the ND LEEF streams.

Stream	Wetted width, *w* (m)	Discharge, Q (L/s)	Regression Slope (k)	Transport Distance, Sw (m)	Uptake Velocity, V_f_ (mm/s)	% Removal	Model R^2^	p-value	
**JUNE–“Day 0”**								** **	** **
SAND	0.5	0.89	NS						
PG	0.5	0.65	NS						
MIX	0.5	0.98	NS						
COBB	0.5	0.76	NS						
**JULY**								** **	** **
SAND	0.56	1.1	-0.017	59	0.024	54%	0.91	0.02	
			(-0.02|-0.013)	(48|79)	(0.018|0.029)				
PG	0.62	0.98	NS						
					
MIX	0.6	1.1	-0.045	22	0.059	88%	0.88	0.04	
			(-0.06|-0.03)	(17|32)	(0.041|0.065)				
			-0.01	130	0.023	8%	0.93	0.02	
COBB	0.59	1.2	(-0.02|-0.014)	(104|172)	(0.018|0.028)				
**AUGUST**								** **	** **
SAND	0.6	1	-0.012	92	0.014	66%	0.74	0.01	
			(-0.02|-0.01)	(52|102)	(0.014|0.020)				
PG	0.62	0.98	-0.053	19	0.068	98%	0.93	0.02	
			(-0.06|-0.041)	(15|24)	(0.053|0.073)				
MIX	0.6	1.16	NS	—	—				
COBB	0.59	1.22	-0.097	10	0.132	98%	0.94	0.01	
			(-0.077|-0.12)	(9|12)	(0.11|0.14)				
**SEPTEMBER**								** **	** **
SAND	0.58	1.11	-0.055	18	0.079	95%	0.94	0.01	
			(-0.07|-0.045)	(15|24)	(0.014|0.093)				
PG	0.65	1.01	-0.009	108	0.012	31%	0.78	0.1	
			(-0.013|-0.005)	(75|109)	(0.007|0.017)				
MIX	0.72	1.24	NS	—	—				
COBB	0.64	1.22	-0.11	9	0.15	98%	0.94	0.01	
			(-0.13|-0.091)	(7|11)	(0.12|0.17)				
**OCTOBER**								** **	** **
SAND	0.48	1.11	-0.021	47	0.03	62%	0.74	0.01	
			(-0.03|-0.01)	(32|80)	(0.014|0.044)				
PG	0.52	1	-0.038	26	0.05	80%	0.95	0.01	
			(-0.05|-0.031)	(22|32)	(0.041|0.059)				
MIX	0.7	1.22	NS	—	—				
COBB	0.68	1.26	-0.12	8	0.163	96%	0.98	0.01	
			(-0.13|-0.11)	(7|9)	(0.15|0.17)				
**POST-DISTURBANCE**								** **	** **
SAND	0.5	0.98	NS	—	—				
PG	0.5	1.09	-0.014	74	0.018	45%	0.79	0.05	
			(-0.02|-0.010)	(51|132)	(0.013|0.049)				
MIX	0.52	1.05	NS	—	—				
COBB	0.55	0.76	NS	—	—				

After converting each regression slope, *k*, to an uptake length (S_w_), we found that average transport distances varied substantially over time and space; measurable *Cry1Ab* uptake lengths ranged from 8–108 m. Across all sampling dates, we found that S_w_ was generally longest in PG and SAND, intermediate in MIX, and shortest in COBB (ANOVA, p > 0.05, Tukey’s HSD p < 0.05, Table 1). Despite the wide range of *Cry1Ab* transport distances measured in our streams, we found that S_w_ did not differ significantly across sampling dates during biofilm colonization (rmANOVA, p > 0.05).

**Fig 4:**
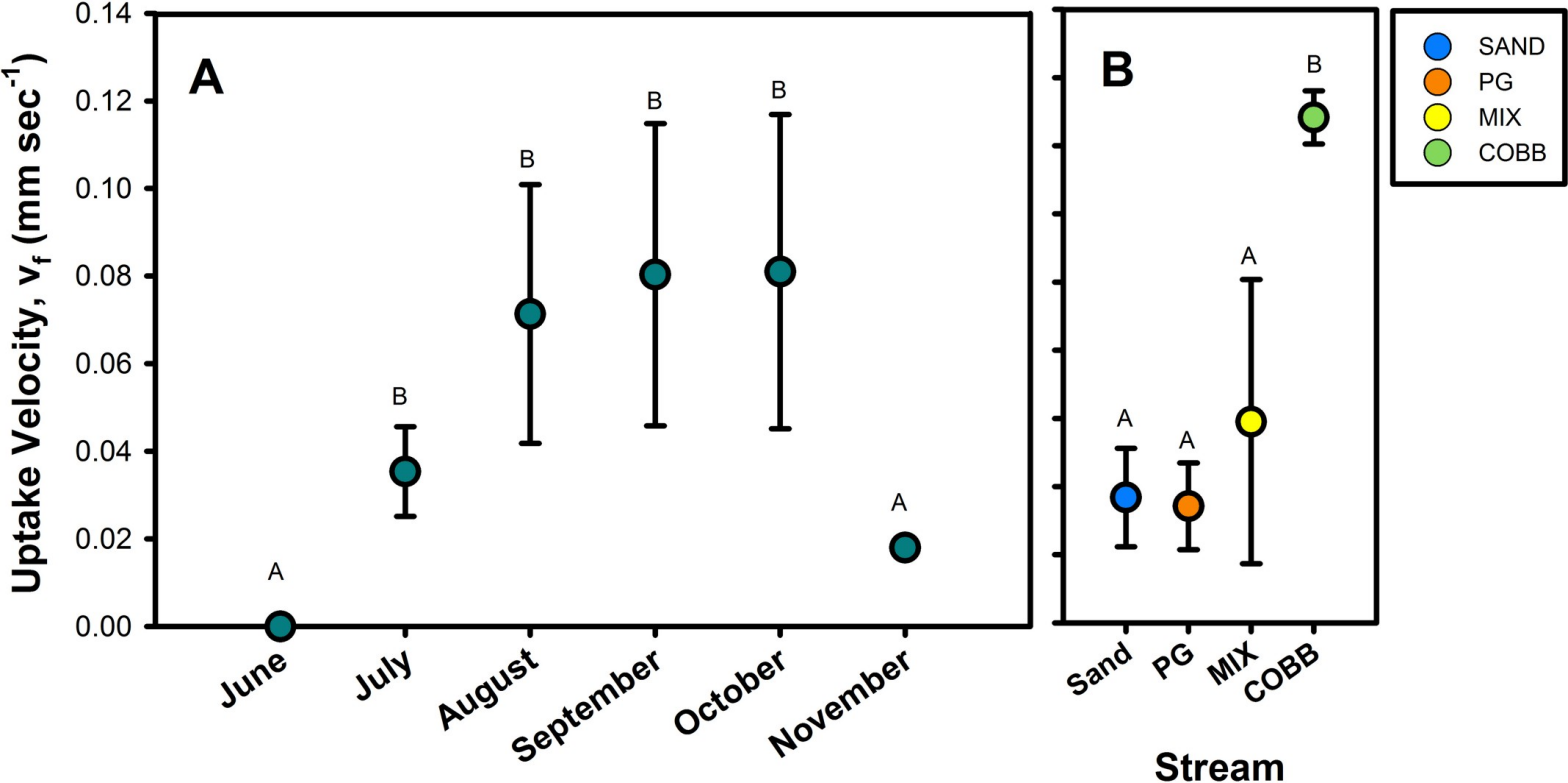
Cry1Ab uptake metrics. *Cry1Ab* uptake velocities (*v*_*f*_) reported as mean (± SE) on A) each sampling date and B) across all sampling days for each stream at ND-LEEF. Upper-case letters denote results of rmANOVA (panel A) and ANOVA (panel B).

Accounting for slight variation in discharge across streams, we found that uptake velocities (as v_f_) for *Cry1Ab* also varied among streams over the sequence of biofilm colonization (Fig 4). Across all sampling dates, v_f_ ranged from no measurable uptake (*June*) to 0.16 mm s^-1^ (*October*) and across all streams averaged 0.06 **±** 0.009 mm s^-1^. *Cry1Ab* v_f_ was significantly different across sampling dates (rm ANOVA, p < 0.01), primarily for the sampling dates during the growth period. Mean uptake measured in June (no detectable uptake) and July were significantly different compared to those measured during peak biofilm in August, September, and October (Tukey HSD, p < 0.01). While v_f_ did not differ statistically among substrate treatments (rm ANOVA, p > 0.05), average v_f_ was highest in COBB and lowest in SAND and PG (Fig 4B).

### How does biofilm colonization influence Cry1Ab uptake metrics?

**Fig 5:**
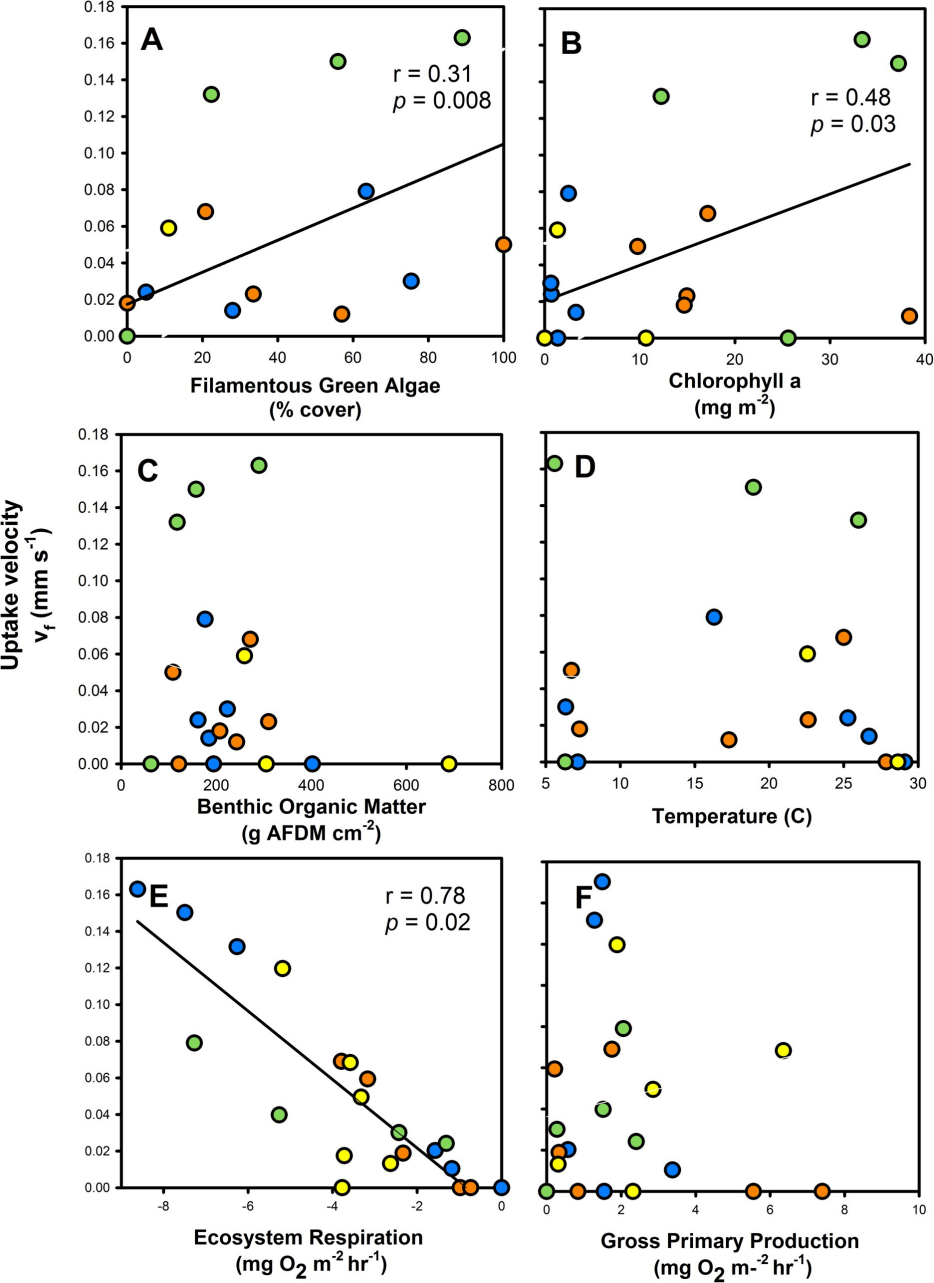
Cry1Ab uptake predictors. Relationships between *Cry1Ab* v_f_ and ancillary variables: A) percent cover filamentous green algae, B) chlorophyll a, C) benthic organic matter, D) temperature, E) ER, and F) GPP. A solid line represents a significant Pearson’s correlation at p < 0.05.

We found significant *Cry1Ab* uptake during times when biofilm had colonized substrate in each stream, suggesting that streams can remove *Cry1Ab* from the water column either via physical retention (e.g., sorption) or via biological removal (e.g., as a DOC source for stream heterotrophs). Across all streams, v_f_ for *Cry1Ab* (mm s^-1^) was related to metrics describing biofilm accumulation, such as % cover of filamentous green algae (LM: R^2^ = 0.31, p = 0.008), chl a (LM: R^2^ = 0.23, p = 0.03) (Fig 5, S1 Table). We also used MLR to determine predictors of v_f_; while the full model, which included water temperature, OM, substrate type, chl a, % cover of filamentous and benthic algae, GPP, and ER, was not significant (LM: R^2^ = 0.77, p = 0.10), the stepwise MLR selected substrate type, biofilm chl a, % cover of filamentous green and benthic algae, and ER as significant controls on v_f_ (LM:R^2^ = 0.94, p < 0.001). We confirmed that biofilm chl a and % cover of filamentous green algae were positively related with *Cry1Ab* v_f_ using Pearson’s correlation analyses (Fig 5, S1 Table). We also found that increasing heterotrophic metabolism (as negative ER) was also correlated with *Cry1Ab* v_f_ (Pearson’s correlation: r = 0.87, p = 0.001). Yet, despite trends with reach-scale metabolism, benthic OM was not a significant predictor of *Cry1Ab* uptake (p > 0.05) (Fig 5). Finally, given the duration of the experiment, spanning multiple seasons, we examined the relationship between v_f_ and water temperature, especially as it declined during the latter part of our experiment. Interestingly, water temperature was not a significant factor in the full linear model (p > 0.05) and was also not correlated with v_f_ (p > 0.05), despite stream temperatures ranging from 5–30°C across from June to November in our study.

The *Cry1Ab* addition conducted immediately following the disturbance event (Day 159 in November) confirmed that biofilm colonization was a significant driver of *Cry1Ab* uptake. We compared these data with results from the release conducted on Day 0 in June, pre-biofilm colonization, when substrate was bare. In both cases, we found no measurable *Cry1Ab* uptake when there was no significant colonization of biofilm (Fig 5).

## Discussion

### Biofilms influenced Cry1Ab uptake

Traditional studies have shown that downstream removal of particles and solutes is influenced by a variety of instream features that promote channel complexity, such as debris dams [29], large woody debris [30], macrophytes [31,32], and benthic biofilms [21,33,34]. More specifically, previous research in the ND-LEEF streams has shown that biofilm colonization increased instream complexity and water residence times [14,35], which promotes nutrient processing via increased biological activity [36].

We predicted that substrate-specific biofilm growth, accumulation, and senescence in the open-canopy experimental streams would influence the fate of *Cry1Ab*, particularly during stable flow during summer and fall. We found that leached *Cry1Ab* uptake velocities (as v_f_) and downstream transport distances (as S_w_) were strongly influenced by biofilm colonization. At the beginning of our experiment, when substrate was still bare, we found no evidence of *Cry1Ab* removal along the stream reaches, meaning that *Cry1Ab* was traveling on average >50 m downstream before removal. However, after biofilm colonization, we were consistently able to measure significant removal within stream reaches, and we found that *Cry1Ab* uptake increased in response to both increases in structural metrics such as biofilm biomass and functional metrics such as ER. We measured highest *Cry1Ab* uptake at the peak of biofilm colonization (v_f_ = 0.03–0.16 mm s^-1^ across all streams), and the average uptake lengths (S_w_) were between 8–47 m. Surprisingly, in contrast to previous studies which found that larger substrate size (i.e., COBB) selected for increased biofilm growth compared to smaller substrate (e.g., PG) [36], we found that substrate had limited effects on biofilm development and *Cry1Ab* removal. Rather, the presence of biofilm, regardless of substrate type, was the most significant predictor of the relative demand for *Cry1Ab* as v_f_. Following the disturbance event, *Cry1Ab* uptake returned to negligible levels resulting from the effective removal of the majority (>80%) of benthic algae and all filamentous green algae, and the corresponding reduction in stream metabolism.

It is important to note that we could not evaluate data statistically when uptake rates were below detection (i.e., slope *k* non-significant), as it is possible that uptake occurred that was simply too low to measure given short reach lengths (50 m). However, it is striking that the only dates on which uptake could not be measured were when there was little to no biofilm, at the start of the experiment and after disturbance. It is likely that on these dates, uptake was minimal due to low biological activity, low biomass, and limited hydrologic exchange with the benthos. However, our observation that uptake could not be detected when biofilm was absent in June and November, while rapid instream removal was measured during July-October when biofilm was growing, supports our finding that biomass accumulation mediates *Cry1Ab* fate.

### Reach-scale Cry1Ab removal

Generally, previous research has shown that *Cry1Ab* proteins do not accumulate in substrate interstices or groundwater [12] and while laboratory studies have shown that *Cry1Ab* degrades quickly (within days) in stream water [10,23]; however, it is not understood how these rates scale in natural systems. To better parse out the relative role of degradation in overall removal, we estimated how far *Cry1Ab* might travel if it was *only* degraded (at rates measured in lab mesocosms) versus if it is both actively retained (i.e., uptake) as well as degraded, combining previously published degradation coefficients (from [10]) and our empirically-derived removal rates (as regression slopes, *k*) from this study.

**Fig 6:**
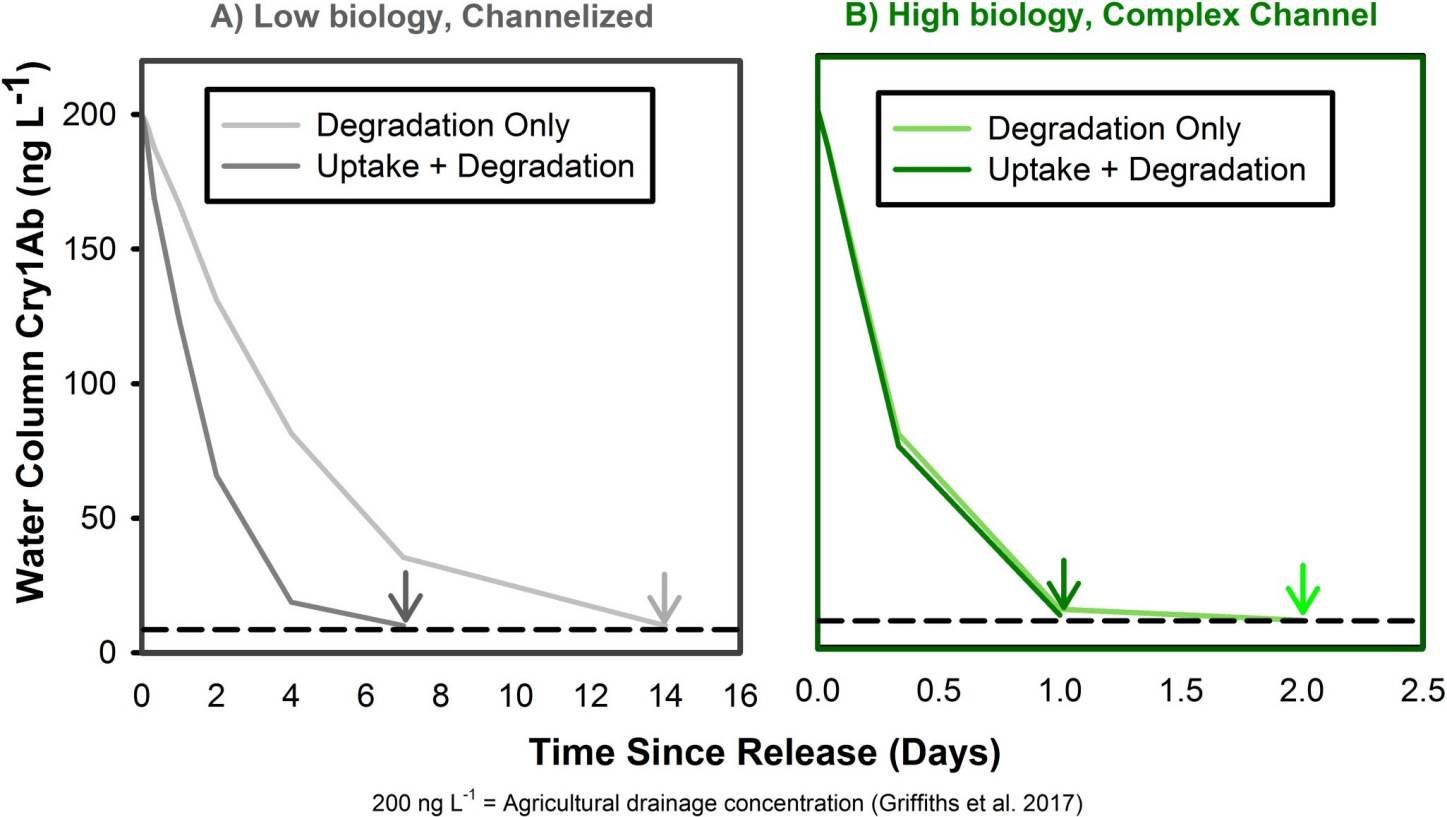
Modelling Cry1Ab degradation and uptake. Modeled “degradation” and “uptake + degradation” scenarios for *Cry1Ab* removal from the water column under A) Low biology in a channelized stream, and B) High biology in a complex channel.

We estimated water column concentrations of *Cry1Ab* over time (at 1, 4, 12 hr, and 1, 2, 7, 14, 30 days) using a simple exponential model to represent *Cry1Ab* removal (C_o_ = C_i_e^-kt^). For the initial concentration (C_o_), we used a starting concentration of 200 ng L^-1^, which represents *Cry1Ab* concentrations from an agricultural drainage in a Midwestern stream [10]. We modeled two scenarios, low biofilm and high biofilm, and projected *Cry1Ab* persistence in time accounting for “degradation only” and “degradation + uptake”. We then compared the projected instream calculation of two “degradation-only” scenarios: Low biofilm (*k*_*D*,*L*_ = -0.119 day^-1^) and High biofilm (*k*_*D*,*H*_ = -1.73 day^-1^) (degradation rates from [10]), to “uptake + degradation” scenarios: Low biofilm (*k*_*R*,*L*_ *=* -0.316) and High biofilm (*k*_*R*,*H*_ *=* -1.84). We found that these alternate biofilm scenarios influence how long *Cry1Ab* might remain detectable in the water column (>10 ng L^-1^ [10]; Fig 6). Generally, for degradation only, water column concentrations would fall below analytical detection limits (10 ng L^-1^) after 2 and 14 days under High and Low Biofilm scenarios, respectively (Fig 6). However, when accounting for the additional role of biological uptake (“degradation + uptake”), parameterized with empirical data from this study, the detection window is reduced to roughly 1 and 6 days for High and Low Biofilm scenarios, respectively (Fig 6). Both our empirical results, and this modelling exercise, suggest that once *Cry1Ab* is leached, high biofilm colonization increases removal and mediates further downstream transport. While the *Cry1Ab* protein is degraded rapidly in the water column, biofilm-mediated removal (i.e., degradation or biological sorption) has significant potential to reduce water column concentrations and attenuate downstream transport of the protein. Our results suggest that *Cry1Ab* detection, removal, and transport, throughout the stream networks may depend strongly on environmental context, via mechanisms occurring in both terrestrial habitats (i.e., agricultural fields) as well as in adjacent waterways.

Because *Cry1Ab* removal is tightly linked to biofilm accumulation, it follows that disturbance events that reduce stream biology could also reduce *Cry1Ab* uptake. In this study, *Cry1Ab* removal was undetectable after all biofilm was manually sloughed from the ND-LEEF streams (Fig 5). Effectively, biofilm removal decreased the uptake capacity of each stream, resulting in longer transport distances, exceeding the length of each stream reach (>50 m). Major disturbances are common in agricultural streams and include dredging, substrate instability [37], and biofilm sloughing from storms [38], all resulting in reduced benthic biofilm complexity, which may lead to diminished removal and increased downstream transport of leached *Cry1Ab*. Additionally, decline in biofilm-mediated uptake following storms could be accompanied by increased inputs of Bt maize detritus from adjacent fields as well as increased *Cry1Ab* leaching from subsurface tile drain flow into streams. These dynamics are intrinsically difficult to capture using simple models and the empirical data presented in this study, but may be important factors when considering the timing and detection of *Cry1Ab* in highly managed ecosystems, such as agricultural streams.

### Implications for Cry1Ab detection in stream networks

Bt maize detritus represents significant reservoir for the *Cry1Ab* protein in the terrestrial environment [5,39]; after harvest, *Cry1Ab* remains detectable within dry detritus for months after harvest which creates the potential for year round leaching of Cry 1Ab to adjacent streams [9,40]. There are multiple pathways by which *Cry1Ab* can enter adjacent waterways including decomposition of buried detritus and pollen deposition [9,39,40]; lateral inputs of dissolved proteins via subsurface tile drainage [5,10,12]; overland flow into surface water [11]; and direct leaching from submerged instream detritus [6,10]. However, previous studies have found that dissolved *Cry1Ab* proteins do not persist long-term in groundwater [12] or in stream water [10,23]. Additionally, surveys of the spatial distribution of dissolved *Cry1Ab* proteins in water samples from Midwestern streams found no obvious spatial aggregation across the landscape [5]. Despite this, the detection of the *Cry1Ab* protein was ubiquitous in waterways suggesting that the source material for the leaching of the protein was widespread, leading to the characterization of *Cry1Ab* as “pseudo-persistent” at the watershed scale [10].

As has been suggested previously [10], there must be a large and consistent source of detrital material in order to sustain detectable *Cry1Ab* concentrations in adjacent freshwaters across the landscape [5]. Here we gain insight on that assumption using a simple budget approach, combining specific details on *Cry1Ab* decomposition [10] with rates of water column removal (*this study*) in order to estimate the size of this source pool. More specifically, using these new estimates, we estimate the detrital biomass of corn necessary to sustain instream concentrations above the minimum streamwater detection of 10 ng/L *Cry1Ab* of leached protein. We started with an estimate of *Cry1Ab* standing stocks for a typical Midwestern watershed, using one we have studied extensively (Shatto Ditch Watershed, IN; drainage area = 12 km^2^) which is drained by a stream with mean annual discharge of 150 L/s. For Shatto, we assumed that 1000 g/m^2^ of detrital corn residue remains on fields in the watershed after harvest [41], that 1g of stover can potentially leach 4.75 μg of *Cry1Ab* (See Fig 2A; [10]), and *Cry1Ab* degrades from harvest in fall through spring planting (6 months) at an approximate rate of -k = 0.021 day^-1^ [41] which results in a minimum of 1.08 mg *Cry1Ab*/m^2^ in the watershed. Given the daily water yield in Shatto of 1.1 L/m^2^/day, and the 6-month exposure period, we estimate 0.6 ug *Cry1Ab* /m^2^/d available to leach, and runoff with a potential concentration of 550 ng *Cry1Ab*/L which is 2–3 orders of magnitude above the environmental detection limit in surface waters. Moreover, it would require only 17.5 g/m^2^ of detrital corn residue to achieve the minimum streamwater detection of 10 ng/L *Cry1Ab* of leached protein. As such, even when considering corn residue degradation, the average mean standing stock of residue far exceeds the minimum necessary for instream detection.

While the on-field calculations above corroborate the large potential source pool of *Cry1Ab* at the watershed-scale, we also need to consider the potential role of instream removal via microbial degradation that we have quantified (*this study*). If *Cry1Ab* runoff enters surface water, how far will it travel before concentrations dip below the stream water detection limit in adjacent surface waters? Using the equation C_i_ = C_o_e^-kx^, the removal rate from this study (k = -0.12 m^-1^), and the above initial concentration of 550 ng *Cry1Ab*/L in runoff, we estimate that the *Cry1Ab* protein would travel only 34 m from its source before dipping below detection. Yet, through field studies, previous research has shown that *Cry1Ab* concentrations in subsurface tile drainage, and receiving waterways, routinely exceed the 10 ng *Cry1Ab*/L detection threshold, with concentrations as high as 200 ng *Cry1Ab*/L [5,10]. We also know that there is high affinity of *Cry1Ab* proteins to sorb to organic matter [42–44] and rapid degradation rates have also been measured in stream water [10,11]. Finally, with this research, we add quantitative estimates of rapid instream removal rates, indicating that stream biofilms, in addition to other removal processes, may create substantial variation in stream concentrations that are “available” for detection. In other words, the concentrations found in stream water may not represent the total mass in a given system. While our study does not address the ultimate fate of these *Cry1Ab* proteins removed by biofilms, it is unlikely that *Cry1Ab* proteins are being transported far from their source. Moreover, the “patchy but ubiquitous” distribution of *Cry1Ab* in agricultural drainage networks [5] reiterates that there must be a very large source pool of detrital maize residue fueling these landscape patterns. Therefore, further exploration of the large potential pool of this novel carbon source, and its influence on adjacent waterways and associated biota, is needed.

### Cry1Ab uptake was comparable to rates measured for DOC

Streams process and transport a variety of terrestrially-derived dissolved organic carbon (DOC) [45], which generally constitutes a complex mixture of compounds. In agricultural streams, leachate from Bt maize, including the *Cry1Ab* protein, represent part of the generalized DOC pool [10]. Short term (i.e., steady state) releases have commonly been used to evaluate the net effect of both biotic and abiotic ecosystem processes on the transport and removal of solutes (e.g., inorganic nutrients, DOC) from the water column at the scale of the stream reach (see review [46]). Extending the application of this method to examine drivers controlling the fate of GE crop byproducts, such as *Cry1Ab*, in aquatic environments is novel, and potentially powerful. In order to evaluate the application of the steady state method to *Cry1Ab* as analogous to commonly used solutes, and to determine whether *Cry1Ab* uptake rates in ND-LEEF streams were comparable to uptake rates for other dissolved organic compounds in natural streams, we compared *Cry1Ab* removal rates to previously published rates of DOC uptake using similar methods (S1 Fig). We found that the *Cry1Ab* uptake measured over our study (mean v_f_ = 0.059 ± 0.009 mm s^-1^) was comparable to uptake for all other DOC forms (v_f_ = 0.034 ± 0.006 mm s^-1^; [46]), and that *Cry1Ab* removal was most similar to previously reported rates simple sugars (v_f_ = 0.049 ± 0.008 mm s^-1^; [10]). We suggest that the *Cry1Ab* uptake rate we measured is likely due to 1) the highly labile nature of *Cry1Ab* or 2) its high affinity for sorption [43], relative to more recalcitrant forms of DOC [47]. In addition, the comparability of *Cry1Ab* uptake rates to DOC validates our use of traditional stream ecology methods for estimating *Cry1Ab* removal and poses a methodological advancement for the continued understanding of this novel and emerging solute in natural waterways. We recognize that our study represents relatively limited temporal and spatial resolution, which may not capture all of the conditions in a natural stream network. However, our results, under even simplified conditions, indicate that environmental heterogeneity likely alters the balance between *Cry1Ab* removal and downstream transport, which has implications for the fate of this protein transported beyond intended boundaries.

## Conclusions

Bt maize detritus is a significant reservoir for *Cry1Ab* proteins in the terrestrial environment [5,39] and agricultural landscapes present many routes of entry to adjacent stream ecosystems. Leached *Cry1Ab* protein from the input of Bt maize debris into streams [3,6,7] or *Cry1Ab* present in runoff or subsurface tile drainage [10] all represent major input pathways of leached GE byproducts into aquatic ecosystems located adjacent to agricultural fields. Once Cry-proteins associated with Bt maize detritus enters streams, it can be processed and transported within stream networks [5,6]. The source of leached *Cry1Ab* protein may be widespread in agricultural streams and ditches, leading to the persistent detection of *Cry1Ab* in watersheds draining agricultural land. Both our empirical measurements and our modeling approach supports the hypothesis that instream removal of *Cry1Ab* may be an important mechanism for removal of leached *Cry1Ab* from the water column, reducing the available pool of the protein susceptible to downstream transport. If stream biofilms rapidly remove *Cry1Ab* and other environmental proteins from the water column, relying solely on instream concentrations may not account for the true pool of Bt maize detritus.

## Supporting information

S1 Table Biofilm statistics. Table of Pearson’s correlations for biofilm characteristics.
(XLSX)

S1 Fig DOC meta-analysis. Placing *Cry1Ab* uptake velocities in context with a previous meta-analysis of DOC v_f_ values (from [46]) with A) median values for individual DOC types and B) mean v_f_ by group.
(TIF)

S1 File All data used in the study are available as a .file. (XLSX)
