# Supplementary figures and images for: Transport and instream removal of the Cry1Ab protein from genetically engineered maize is mediated by biofilms in experimental streams
Source: PLoS One. 2019 May 16;14(5):e0216481. doi: 10.1371/journal.pone.0216481 (PMC6522009; doi:10.1371/journal.pone.0216481)

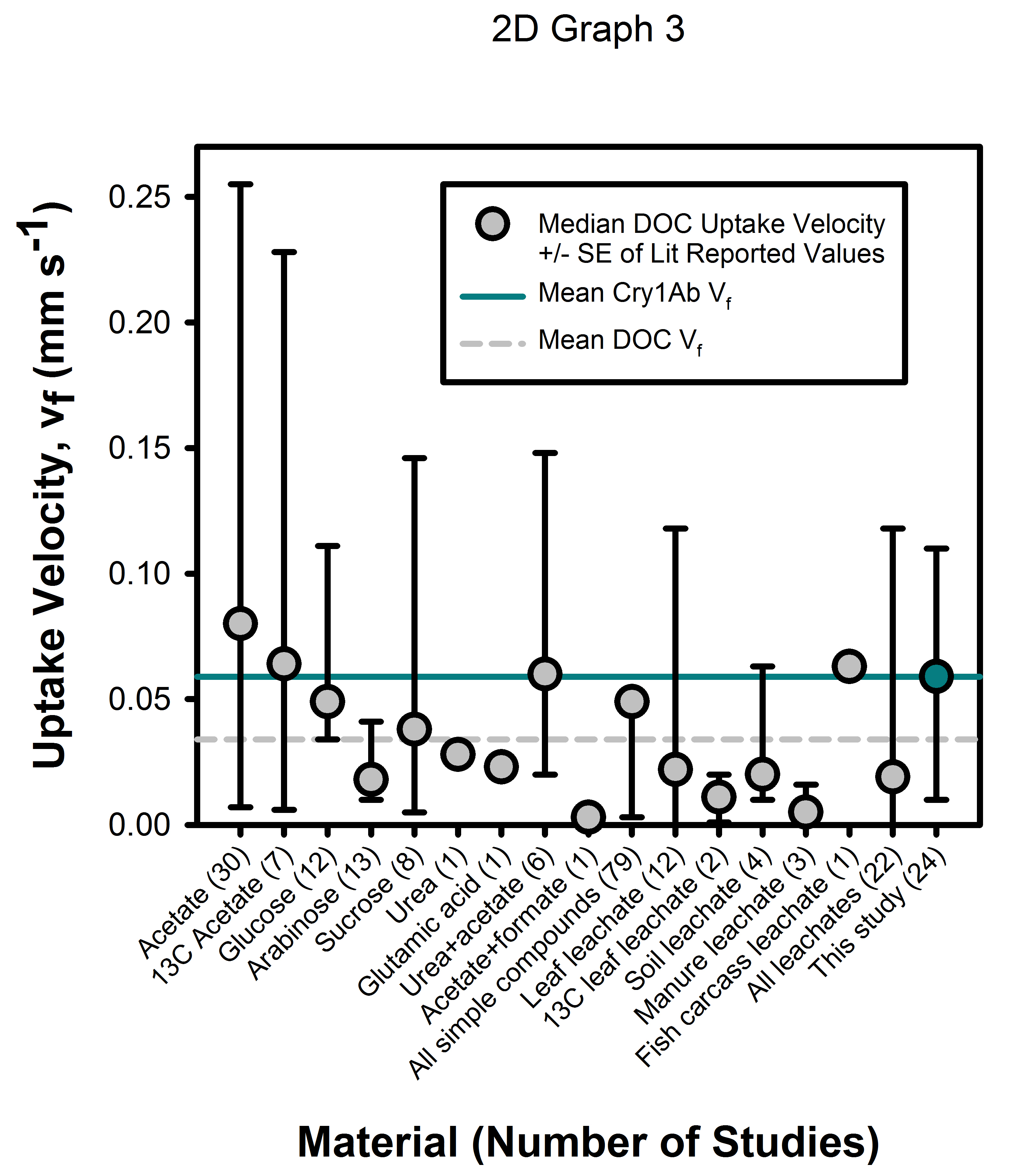

Supplement: S1 Fig — Placing Cry1Ab uptake velocities in context with a previous meta-analysis of DOC vf values (from [46]) with A) median values for individual DOC types and B) mean vf by group. (TIF) [file pone.0216481.s002.tif]
